# FurIOS: A Web-Based Tool for Identification of *Vibrionaceae* Species Using the *fur* Gene

**DOI:** 10.3389/fmicb.2017.00414

**Published:** 2017-03-13

**Authors:** Henrique Machado, João Cardoso, Sonia Giubergia, Kristoffer Rapacki, Lone Gram

**Affiliations:** ^1^Department of Biotechnology and Biomedicine, Technical University of Denmark, Kongens LyngbyDenmark; ^2^The Novo Nordisk Foundation Center for Biosustainability, Technical University of Denmark, Kongens LyngbyDenmark; ^3^Center for Biological Sequence Analysis, Department of Bioinformatics, Technical University of Denmark, Kongens LyngbyDenmark

**Keywords:** *Vibrionaceae*, *Vibrio*, *Photobacterium*, *fur* gene, phylogeny, identification

## Abstract

Gene based methods for identification of species from the *Vibrionaceae* family have been developed during the last decades to address the limitations of the commonly used 16S rRNA gene phylogeny. Recently, we found that the *ferric-uptake regulator* gene (*fur*) can be used as a single identification marker providing species discrimination, consistent with multi-locus sequencing analyses and whole genome phylogenies. To allow for broader and easy use of this marker, we have developed an online prediction service that allows the identification of *Vibrionaceae* species based on their *fur*-sequence. The input is a DNA sequence that can be uploaded on the web service; the output is a table containing the strain identifier, *e*-value, and percentage of identity for each of the matches with rows colored in green for hits with high probability of being the same species. The service is available on the web at: http://www.cbs.dtu.dk/services/furIOS-1.0/. The *fur*-sequences can be derived either from genome sequences or from PCR-amplification of the genomic region encoding the *fur* gene. We have used 191 strains identified as *Vibrionaceae* based on 16S rRNA gene sequence to test the PCR method and the web service on a large dataset. We were able to classify 171 of 191 strains at the species level and 20 strains remained unclassified. Furthermore, the *fur* phylogenetics and subsequent *in silico* DNA-DNA hybridization demonstrated that two strains (ATCC 33789 and ZS-139) previously identified as *Vibrio splendidus* are more closely related to *V. tasmaniensis* and *V. cyclitrophicus*, respectively. FurIOS is an easy-to-use online service that allows the identification of bacteria from the *Vibrionaceae* family at the species level using the *fur* gene as a single marker. Its simplistic design and straightforward pipeline makes it suitable for any research environment, from academia to industry.

## Introduction

A key aspect in microbial taxonomy is the identification of microorganisms at the species or genus level. This is important to distinguish pathogenic species in health and environmental sciences, to identify beneficial or symbiotic species and also to study microbial diversity in environmental niches in Nature. Due to the widespread use and importance of bacterial identification, methods and processes should be accurate, affordable, fast and easy to use (Urakawa et al., 1997; Amaral et al., 2014).

The *Vibrionaceae* is a large family of marine Gram-negative *Gammaproteobacteria*, which includes organisms of different environmental importances, e.g., symbiotic (e.g., *Vibrio fischeri*), bioactive (e.g., *V. coralliilyticus* and *Photobacterium galatheae*), and pathogenic organisms (e.g., *V. cholerae* and *Aliivibrio salmonicida*). Several of the human pathogenic *Vibrio* species, such as *V. cholerae* and *V. parahaemolyticus*, are mesophilic organisms and changes in sea water temperature and salinity influence their distribution (Reid et al., 2011; Huehn et al., 2014; Le Roux et al., 2015; Xu et al., 2015). The increase in sea water temperature has been linked to a number of human illness outbreaks caused by *Vibrio* species (e.g., *V. cholerae*, *V. parahaemolyticus*, *V. vulnificus*) (Le Roux et al., 2015) and to several epidemics in marine animals, such as oysters, salmon, sea bass, eel, trout, shrimps, and corals, caused by *V. salmonicida*, *V. anguillarum*, *P. damselae*, *V. vulnificus*, and *V. coralliilyticus* (Ottaviani et al., 2012; Huehn et al., 2014; Le Roux et al., 2015). The rising sea water temperatures have also been linked to the increase in the numbers of *V. cholerae* associated with plankton in the North Sea (Vezzulli et al., 2012). The increased spread of *Vibrio* pathogens and their importance as infectious and/or food poisoning agents has a direct impact on health of mankind and requires changes in microbiological food control processes and clinical settings (Nair et al., 2007). Any survey or intervention to reduce risk requires that the organisms can be rapidly and correctly identified.

Identification of species from the *Vibrionaceae* family has primarily been based on Multi-Locus Sequencing Analysis (MLSA), which relies on the amplification and sequencing of up to nine genes (*ftsZ*, *gapA*, *gyrB*, *mreB*, *pyrH*, *recA*, *rpoA*, *topA*, and the 16S rRNA gene) (Thompson et al., 2005; Sawabe et al., 2007, 2013; Gabriel et al., 2014). This has been necessary due to the limitations of 16S rRNA gene phylogeny in this family, an issue addressed with coupling of other techniques such as restriction fragment length polymorphism analyses (Urakawa et al., 1997, 1999), but ultimately unsolvable due to several (7–15) different copies of 16S rRNA gene encoded in a single genome (Reen et al., 2006; Jensen et al., 2009; Machado and Gram, 2015). We have recently identified the *ferric-uptake regulator* gene (*fur*) as a new phylogenetic marker in the *Vibrionaceae* family, and developed a PCR based method for the amplification of the genomic region encoding the *fur* gene (Machado and Gram, 2015). Its use as a single phylogenetic marker in the classification of *Vibrionaceae* at the species level could reduce the timing and cost of strain identification. To enable the use of this potential worldwide, we here describe the design of an online platform, FurIOS 1.0, which allows an easy and fast identification of *Vibrionaceae* species using only their *fur* DNA sequence. We also use a collection of 191 *Vibrionaceae* strains to demonstrate its potential.

## Materials and Methods

### Database Design

A *fur* gene sequences database was created by extracting the sequences from available whole genome sequences and using the data collected during the design of the gene amplification methodology (Machado and Gram, 2015). The BLAST compatible version was generated using *makeblastdb* from NCBI BLAST+ command line tools.

### Implementation of FurIOS 1.0

FurIOS is implemented in Python, compatible with version 2. The BLAST is performed using NCBI BLAST+ version (2.2.28+) (Tatusova and Madden, 1999), *blastn* calls and output parsing is handled using BioPython (Cock et al., 2009). The script verifies the format of the input, parameterizes the *blastn* command call and formats the output. BLAST runs with the following parameters: *e*-value of 10; gap open penalty of 5; gap extension penalty of 2; mismatch penalty of -3; match reward of 2; word size of 11; maximum number of returned alignments of 50. These parameters are the default CLC Main Workbench (CLC Aarhus, Denmark version 7) used in the design of the method and evaluation of the potential of the *fur* as a phylogenetic marker (Machado and Gram, 2015). The web interface is provided by the Center for Biological Sequence Analysis (CBS), Technical University of Denmark and the webpages are designed according to their standards. Documentation can be also found on the website.

### Testing of the Identification Pipeline

#### Bacterial Strains and Genomic DNA Extraction

The bacterial strains used in the implementation of the identification pipeline were 191 *Vibrionaceae* strains from the Galathea 3 culture collection (Gram et al., 2010). These strains have been identified as *Vibrionaceae* by analysis of the 16S rRNA gene sequence (Gram et al., 2010). Here, we aimed at a more specific classification, at the species level. Strains were grown overnight at 25°C and 200 r.p.m. in Marine Broth (Difco 279110), before genomic DNA was extracted using the NucleoSpin^®^ Tissue Kit (Macherey-Nagel, Düren, Germany). Genomic DNA quality was checked by 1% agarose gel electrophoresis and quantified by absorbance using DeNovix DS-11 (DeNovix Inc., Wilmington, DE, USA).

#### PCR Amplification and Sequencing

Amplification of the *fur* gene was performed as previously described (Machado and Gram, 2015), with few changes. Briefly, amplifications were performed in a total volume of 25 μL using 5 ng final concentration of genomic DNA as template, 0.2 μM of each primer [fur_AP_fw and fur_AP_rv (Machado and Gram, 2015)] and TEMPase Hot Start Master Mix Blue (Ampliqon A/S, Odense, Denmark), following the producers instructions. The PCR amplification was carried out in a thermal cycler (Applied Biosystems^®^ Veriti^®^ 96-Well Thermal Cycler) as follows: 15 min initial denaturation step at 95°C, followed by 30 cycles of 95°C for 25 s, 52°C for 25 s, and 72°C for 1 min, with a final extension step of 5 min at 72°C. The amplified products were visualized after agarose gel electrophoresis (1%) and ethidium bromide staining. The PCR products were enzymatically purified by treatment with Exonuclease I (ExoI) (Thermo Scientific) and FastAP Thermosensitive Alkaline Phosphatase (Thermo Scientific) before being sequenced by Macrogen (Macrogen Europe, Amsterdam, The Netherlands). The sequencing reads were assembled and analyzed using CLC Main Workbench (CLC Aarhus, Denmark version 7).

#### Species Identification

The *fur* gene sequences obtained from the PCR and sequencing reactions were merged into a single FASTA file. This file contained 191 *fur* sequences, each with a headline identifying the strain number (e.g., “>S2757”). This file was uploaded to the web interface of the FurIOS 1.0^1^ and the analyses performed. The highest hit was selected from the output for each of the strains (Supplementary Table S1) and the strain classified at the species level if the percentage of identity was above 95%. In cases where lower than 95% identity was obtained, the strain was classified as a possible new species. The *fur* gene sequences have been deposited on GenBank under accession numbers KU756296 – KU756481, KP721394, KP721390, KP721391, KP721399, and KP721400.

### Genomic Analyses of *Vibrio splendidus* Strains

*In silico* DNA-DNA hybridization was performed for *V. splendidus* strains representing three different *fur* phylogenetic clusters. The genomes were compared using the Genome-to-Genome Distance Calculator 2.1 (GGDC) tool from DSMZ^2^ (Auch et al., 2010a,b; Meier-Kolthoff et al., 2013). The used whole genome sequences of *V. splendidus* strains FF-500, 1F-157, FF-6, ZF-90, 1S-124, ZS-139, ATCC 33789, and NCCB 53037^T^ are publically available at NCBI under whole genome sequenced accession numbers AJZH00000000, AJZJ00000000, AJZI00000000, AJZF00000000, AJZL00000000, AJZE00000000, AFWG00000000, LNQX00000000, respectively.

## Results

### Database Design and Implementation

The designed database includes 134 sequences representing 78 species of the *Vibrionaceae* family, covering its six genera (*Vibrio*, *Photobacterium*, *Aliivibrio*, *Grimontia*, *Enterovibrio*, and *Salinivibrio*). This includes whole the available *fur* sequences, weather individual or retrieved from whole genome sequences.

**FIGURE 1:**
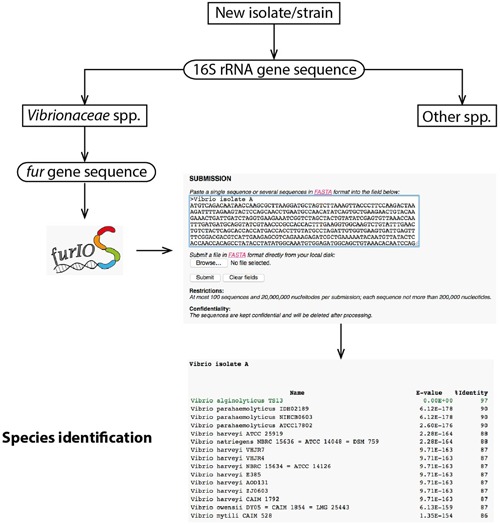
**Workflow and visualization example.** Example of sequence submission, this should be in FASTA format and can be a multiple sequence submission. Example of a result in table format, which includes the *E*-value and the percentage of identity; the predicted species is colored in green.

Users can access FurIOS via the web interface: http://www.cbs.dtu.dk/services/furIOS-1.0/. The input can be the whole *fur* open-reading frame of the sequence of the strain to be identified, the whole sequenced fragment, whole genome sequences or metagenomics data (with a maximum of 100 contigs of up to 200,000 nucleotides, making a total of 20 million nucleotides per submission). Submission of untreated sequences (raw sequencing data) is possible and decreases the need for sequence processing by the user, thereby accelerating the identification process. The sequences can be uploaded on the web service by “copy and paste” or using a file in FASTA format (**Figure 1**). The output is a table containing the strain identifier, *e*-value, and percentage of identity for each of the matches with rows colored in green for hits with percentage of identity higher than 95% (**Figure 1**), representing a high probability of being the same species (Machado and Gram, 2015). The service is provided with pre-established settings used in the design and evaluation of *fur* as a phylogenetic marker (Machado and Gram, 2015), however, a portable version where parameters can be selected by the user will be provided upon request.

### Testing of the Identification Pipeline

The identification pipeline from isolate to species identification was used in the classification of the *Vibrionaceae* isolates from the Galathea 3 culture collection (Gram et al., 2010). These strains have been previously identified as *Vibrionaceae* by 16S rRNA gene sequence analysis (Gram et al., 2010). Here, we aimed at a more specific classification, at the species level. Therefore, genomic DNA isolation followed by *fur* gene amplification and sequencing was performed in 191 *Vibrionaceae* strains.

**FIGURE 2:**
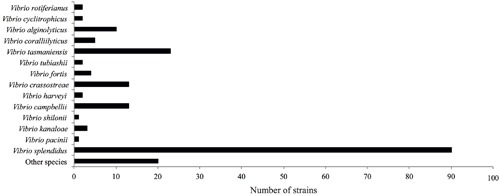
**Classification of 191 strains belonging to the Galathea 3 *Vibrionaceae* culture collection (Gram et al., 2010).** The identification corresponds to the highest hit obtained from the analyses using FurIOS 1.0. In cases where lower than 95% identity was obtained, the strain was classified as “other species.”

**FIGURE 3:**
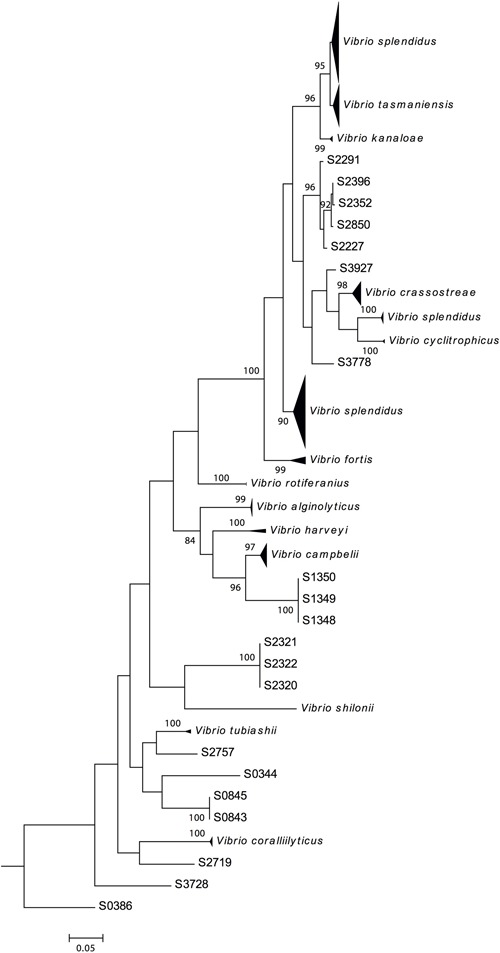
**Phylogenetic tree of 191 Galathea 3 *Vibrionaceae* strains.** The tree is based on the complete *fur* gene sequences analysis and was constructed by the neighbor-joining method. S0386 was used as the outlier, since it classified as a *Photobacterium* sp. The nodes with bootstrap support of 70 or more are indicated (1000 replications).

The *fur* gene sequences obtained were analyzed using FurIOS 1.0. Species affiliation could be assigned to 171 of the 191 strains (**Figure 2**). Based on the *fur* sequence, the remaining 20 isolates had an identity lower than 95% and were therefore classified as “other species” (**Figure 2**). These 20 strains could potentially be new *Vibrio* species or species not yet represented in the FurIOS database, due to unavailability of whole genome or *fur* sequences. Of the 20 “other species,” 15 were 90–94% similar to the *fur* gene sequences of known species, whereas the remaining five strains had similarities between 82 and 89% (Supplementary Table S1). From the phylogenetic analysis it is also possible to evaluate the relatedness of some of the strains identified as “other species” (**Figure 3**). Some of these will most likely be the same species (e.g., S1348, S1349, and S1350, or S2320, S2321, and S2322).

### Genomic Analyses of Vibrio splendidus Strains

Ninety of the 171 strains were identified as *V. splendidus*, however, these did not cluster as one tight single cluster but were distributed across the phylogenetic tree (**Figure 3**). This species was represented in three clusters, two large and one small, all placed within the *Splendidus* clade, to which *V. fortis*, *V. cyclitrophicus*, *V. crassostreae*, *V. tasmaniensis*, and *V. kanaloae* species also belong (Sawabe et al., 2013). The three *V. splendidus* clusters correspond to *fur* homology to different *V. splendidus* strains. The first cluster contained strains with *fur* homology to *V. splendidus* strains FF-6, FF-500, 1F-157, 1S-124, and ZF-90 (Supplementary Table S1). The second smaller cluster and the third cluster contained strains with *fur* homology to *V. splendidus* ZS-139 and ATCC 33789, respectively. The different clustering suggests a different phylogenetic relationship between these strains, previously identified as the same species.

These discrepancies have been previously reported and attributed to the possible misidentification of *Vibrio* strains (Gomez-Gil, 2004; Thompson et al., 2007; Lin et al., 2010) or to the genetic diversity and polyphyletic nature of *V. splendidus* (Thompson and Hoste, 2001; Thompson et al., 2005; Pascual et al., 2010). To address this, *in silico* DNA-DNA hybridization was performed for strains of the *V. splendidus* representing the three different *fur* phylogenetic clusters of this species. Based on DNA-relatedness (**Table 1**), strains ATCC 33789 and ZS-139 were not similar enough to *V. splendidus* type-strain NCCB 53037^T^ to be considered the same species. These possibly represent a new *Vibrio* species. The *fur* sequences with high homology to the sequence from strain ZS-139 were closely related to *V. cyclitrophicus* (93% identity) while homology to strain ATCC 33789 placed them phylogenetically close to *V. tasmaniensis* (91% identity).

**Table 1:** *In silico* DNA-DNA hybridization estimate of the different *Vibrio splendidus* strains.

	DDH-estimate (%)							
	FF-500	1F-157	FF-6	ZF-90	1S-124	ZS-139	ATCC 33789	NCCB 53037^T^
NCCB 53037^T^	72.20% ± 2.92	70.20% ± 2.92	70.40% ± 2.92	69.50% ± 2.92	69.50% ± 2.92	35.10% ± 2.48	28.00% ± 2.43	100% ± 0.00
ATCC 33789	28.30% ± 2.43	28.30% ± 2.43	28.10% ± 2.43	28.10% ± 2.43	28.10% ± 2.43	27.30% ± 2.43	100% ± 0.00	
ZS-139	35.30% ± 2.48	35.40% ± 2.48	35.00% ± 2.48	35.20% ± 2.48	35.50% ± 2.48	100% ± 0.00		
1S-124	70.20% ± 2.92	72.30% ± 2.92	73.00% ± 2.92	71.00% ± 2.93	100% ± 0.00			
ZF-90	70.20% ± 2.92	71.20% ± 2.93	71.00% ± 2.93	100% ± 0.00				
FF-6	71.20% ± 2.93	71.10% ± 2.93	100% ± 0.00					
1F-157	70.60% ± 2.93	100% ± 0.00						
FF-500	100% ± 0.00							

*The estimate was performed using the Genome-to-Genome Distance Calculator 2.1 (GGDC) tool from DSMZ.*

## Discussion

FurIOS is an easy-to-use online service that allows the identification of bacteria from the *Vibrionaceae* family at the species level using the *fur* gene as a single identification marker. This online service available to any user worldwide is an extra effort to implement the use of the recently developed method for the amplification of the *fur* gene sequence, with greater discriminatory power when compared to MLSA or 16S rRNA analyses. Here, we have applied this tool in the classification of environmental *Vibrionaceae* strains belonging to the global culture collection Galathea 3.

We identified at the species level 90% of the 191 *Vibrionaceae* strains from the Galathea 3 culture collection. The most abundant species was *Vibrio splendidus*, followed by *V. tasmaniensis*, *V. crassostreae*, and *V. campbellii* (**Figure 2**). The collection was based on culturing from marine samples and subsequent testing of antibacterial activity against the fish pathogen *V. anguillarum* (Gram et al., 2010), and this may explain the over-representation (70%) of the species belonging to the *Splendidus* clade (Sawabe et al., 2013). Several studies have shown the presence of *V. splendidus* in water samples through all seasons, with higher predominance in summer, and in locations ranging from the arctic to the tropics (Thompson et al., 2004; Jensen et al., 2009). These features reflect the great adaptability of this species (Jensen et al., 2009), which can also explain its over-representation in a global culture collection such as the Galathea 3 collection. The analysis also provided evidence that two *V. splendidus* strains ATCC 33789 and ZS-139 are indeed not *V. splendidus* species, which we confirmed using whole genome phylogenetic analysis (**Table 1**).

Besides the over-representation of strains from the *Splendidus* clade, this dataset allowed the evaluation of the potential of this identification method and web-tool in the classification of *Vibrionaceae* environmental samples at the species level, by means of a single PCR reaction and sequencing of its product. The method presented here can be used for identification of *Vibrionaceae* species in less than 24 h, and it requires minimal equipment and service. The analyses of the 191 sequences using the FurIOS web service took only 3 min, although this time may depend on the server usage at the moment of use. Sequence based identification has in other settings also been developed into online tools, which have been successfully used for several years for MLSA of pathogenic bacteria^3^,^4^,^5^. A tool based on MLSA has been under development for identification of *Vibrio* species^6^, however, it appears not to be functional. FurIOS is therefore the first functional online service developed for the classification of *Vibrionaceae* species and it is easier to implement and use than the MLSA based analyses, because it uses a single gene with great discriminatory power (Machado and Gram, 2015; Giubergia et al., 2016).

This identification exercise provided 20 isolates with an inconclusive species attribution (“other species” – **Figure 2**). These might constitute new species or species not yet represented in the FurIOS database. Although the number of species represented in the database is lower than the number of species so far described within this family, the use of the *fur* gene as an identification marker and the publication of more *fur* sequences will allow the improvement of the database by increasing the number of species represented. Newly published *fur* sequences can be sent to the corresponding author who will act as a database curator. Also publications making use of FurIOS will be checked for relevant sequences to be added. Supporting the possibility of “other species” being a new species is the case of strain S2757. Its *fur* gene sequence had an 89% identity to *V. tubiashii* DSM 19142 and it was therefore here classified as “other species.” This strain has been further studied and has been recently described as the type strain of the new species *V. galatheae* (Giubergia et al., 2016). In that study, 16S rRNA, MLSA, and *fur* gene analyses were performed and the *fur* gene, as a single gene analysis, provided as good discrimination as did the MLSA analysis (Giubergia et al., 2016). This exemplifies the use of the *fur* gene in the classification of a new species belonging to the *Vibrionaceae* family.

Although there might not be a perfect single gene for species identification or phylogenetic evaluation, the *fur* gene seems so far the best “single” gene identified in the classification of *Vibrio* species and the development of this tool is an effort to bring that to use and help the scientific community with identification of strains from the *Vibrionaceae* family. Yet, evaluation of specific phylogenetic relationships should be done using multiple genes, such as MLSA analyses. It is not clear why *fur* mutational rate allows distinction of *Vibrionaceae* at the species level, but we have observed indications of the same pattern in *Pseudoalteromonas* (Machado et al., 2015).

The recent findings have provided the *Vibrionaceae* taxonomy field with new and more accurate approaches to evaluate the phylogeny and taxonomy relatedness between the different species of this family. This comes as a great opportunity to re-evaluate the evolutionary theories in this family as well to discover and correctly classify newly identified species. Regarding FurIOS, its simplistic design and straightforward pipeline makes it suitable for any research environment, from academia to industry, and especially for quick species identification in clinical and food-safety settings, where *Vibrionaceae* strains are of high risk (e.g., microbiological control of sea food products).

## Author Contributions

HM designed the study and the database used, and together with SG performed the testing of the web-based tool. JC carried out the programming and together with KR the implementation of the web-based tool. All authors contributed to the writing of the manuscript. All authors read and approved the final manuscript.

## Conflict of Interest Statement

The authors declare that the research was conducted in the absence of any commercial or financial relationships that could be construed as a potential conflict of interest.

## Footnotes

1 http://www.cbs.dtu.dk/services/furIOS-1.0/

2 http://ggdc.dsmz.de/

3 http://mlst.warwick.ac.uk/mlst/

4 http://www.mlst.net/

5 http://bigsdb.web.pasteur.fr/

6 http://www.lge.ibi.unicamp.br/temp/vibrionaceae/
